# Evaluation of COVID-19 based on ACE2 expression in normal and cancer patients

**DOI:** 10.1515/med-2020-0208

**Published:** 2020-07-07

**Authors:** Peng Ren, Caifeng Gong, Shaohua Ma

**Affiliations:** Department of Thoracic Surgery, Peking University Third Hospital, Beijing, 100191, China; Department of Oncology, National Cancer Center, National Clinical Research Center for Cancer, Cancer Hospital, Chinese Academy of Medical Sciences and Peking Union Medical College, Beijing, 100021, China

**Keywords:** SARS-CoV-2, ACE2, COVID-19, malignant tumor, ARDS

## Abstract

Severe acute respiratory syndrome coronavirus 2 (SARS-CoV-2) infection is now a serious public health problem. Angiotensin-converting enzyme 2 (ACE2) recognized as the receptor of SARS-CoV is also necessary for SARS-CoV-2. However, the impact of ACE2 on SARS-CoV-2 susceptibility and the situation of malignant tumor patients in this outbreak are unclear. So, it is important to understand the expressions of ACE2 in different normal tissues and cancers. The results showed that the kidneys, duodenum, intestine, gallbladder and testis had the highest ACE2 expressions, followed by the colon, rectum and seminal vesicles. The lungs had a very low expression. ACE2 expressions were upregulated in renal cancer, gastrointestinal tumor and lung cancer. ACE2 expression levels may affect SARS-CoV-2 infection and severity. A total of 3,421 cases with COVID-19 have been collected. Among them, 43 cases (1.26%) had malignant tumor coexisting conditions. The rate of severe events for malignant tumor patients was 39.02% (16/41), while the rate of severe events for all patients was 10.79% (194/1,798). The clinical symptoms and signs were studied for the following three systems: respiratory (31–92%), digestive (10–13%) and urinary systems (3.38%). It seems that symptom severity is not related to protein expression levels. This might be the reason for SARS-CoV-2 showing higher regeneration index and susceptibility. More research is needed to explore the mechanisms and treatments.

## 1 Introduction

Several unexplained pneumonia cases were reported in December 2019, caused by a novel coronavirus [1,2,3]. Patients presented with fever, dry cough, weakness and dyspnea, and some patients were seriously ill with acute respiratory distress syndrome (ARDS) or even died. The International Committee on Taxonomy of Viruses named the virus severe acute respiratory syndrome coronavirus 2 (SARS-CoV-2). By April 26, 2020, the rapid spread of the virus resulted in more than 2.8 million cases and more than 1,90,000 deaths worldwide, and cases have been reported in 211 countries (including the United States, Italy and Spain). The WHO has announced that the 2019 new coronavirus disease (COVID-19) caused by SARS-CoV-2 is a public health emergency of international concern (https://covid19.who.int/). Angiotensin-converting enzyme 2 (ACE2) has been first implicated in heart functioning, hypertension and diabetes, with its effects being mediated, in part, through its ability to convert angiotensin II to angiotensin-1 to 7 [4]. Unexpectedly, previous studies on SARS-CoV have shown that ACE2 was a receptor through which the virus enters cells [5]. Genetic analysis of SARS-CoV-2 has shown that this new virus has approximately 80% sequence identity with SARS-CoV, and further results indicated that SARS-CoV-2 was likely to also use the same receptor ACE2 as the entry receptor [6,7,8,9]. The expression of ACE2 in different tissues is closely related to the susceptibility to and severity of the virus infection [10,11].

Studies have reported that organ dysfunctions such as shock, ARDS, acute cardiac injury, acute kidney injury (AKI) and death can occur in severe cases of COVID-19 [12,13]. More clinical data analysis showed that severe infections were more likely to affect older men with comorbidities such as hypertension, diabetes, cardiovascular disease and cerebrovascular disease [14]. Malignant tumor is one of the most common diseases affecting people’s survival status. These patients will have pathological changes in the body, especially in the immune system. As the incidence of tumors is getting higher and higher, it is now developing chronically and has become the common comorbidity of COVID-19. However, the current situation of tumor patients in this outbreak has not been clearly reported in detail. Therefore, we would like to evaluate the differences in ACE2 expression in various tissue types and cancers. Then, the influence of these differences on the impact of SARS-CoV-2 infections was analyzed. In addition, tumor patients with COVID-19 were briefly evaluated to assess the susceptibility and severity.

## 2 Materials and methods

### 2.1 Data source

The genomic location of the ACE2 gene was downloaded from GeneCard (https://www.genecards.org). COMPARTMENTS was used for gene subcellular location confidence analysis. The mRNA and protein expressions of ACE2 in different normal tissues were obtained from the Human Protein Atlas (HPA). Gene expressions of ACE2 were verified using Genotype Tissue Expression (GTEx) projects. Comparison of the expressions between tumors and normal tissues was performed using the Gene Expression Profiling Interactive Analysis (GEPIA) dataset and ONCOMINE. Protein expression levels and immunohistochemistry (IHC) images were obtained from the HPA. Clinical data in published references for COVID-19 (Coronavirus Disease 2019) patients infected with SARS-CoV-2 were collected. All datasets and clinical data were retrieved from the published literature, so it was confirmed that written informed consent was obtained. Therefore, approval from local ethics committee is not required for this study.

### 2.2 COMPARTMENTS localization

COMPARTMENTS localization data are integrated from literature manual curation, high-throughput microscopy-based screens, predictions from primary sequences and automatic text mining (see COMPARTMENTS: unification and visualization of protein subcellular localization evidence). Unified confidence scores of the localization evidence are assigned based on evidence type and source. The results were visualized in the schematic cell image.

### 2.3 GEPIA analysis

ACE2 mRNA expressions in tumors and normal tissues were obtained from the GEPIA dataset (http://gepia.cancer-pku.cn). It is a newly developed interactive web server for analyzing the RNA sequencing expression data of 9,736 tumors and 8,587 normal samples from the Cancer Genome Atlas and GTEx projects, using a standard processing pipeline. ACE2 expressions of samples in survival analysis were divided into two groups using median expression (high vs low expression) and assessed using a Kaplan–Meier survival plot, with the log-rank test. Significant difference was regarded as *P* < 0.05.

### 2.4 ONCOMINE analysis

ONCOMINE gene expression array datasets (https://www.oncomine.org), an online cancer microarray database, were used to analyze the gene expression of ACE2 in different cancers. The expressions of ACE2 in clinical cancer specimens were compared with those in normal controls, using Student’s *t* test to generate a *P* value. The threshold settings were as follows: *P* value: 1 × 10^−4^; fold change: 2; gene rank: top 10%; and data type: all.

### 2.5 HPA

The mRNA and protein expressions of ACE2 were identified differentially in human normal and cancer tissues using the HPA, a website (https://www.proteinatlas.org) that contains IHC-based expression data for approximately 20 most common types of cancers, with 12 individual tumors in each cancer. ACE2 mRNA levels of different normal tissues were obtained from the Consensus dataset. Consensus Normalized eXpression levels for 55 tissue types and 6 blood cell types were created by combining the data from three transcriptomics datasets (HPA, GTEx and FANTOM5) using the internal normalization pipeline. Protein expression data are shown for each of the 44 normal tissues. Renal cancer, colorectal cancer and lung cancer along with kidney, colon and lung normal tissues with IHC staining of ACE2 protein were chosen with antibody CAB026174.

### 2.6 System review of malignant tumors in COVID-19 patients infected with SARS-CoV-2

Studies recently published describing the clinical characteristics of COVID-19 due to SARS-CoV-2 were screened and analyzed. Studies describing patients’ malignant tumor status were enrolled. The number of patients and severity rate combined with malignant tumors were calculated. Clinical symptoms and signs due to COVID-19 were collected. Studies with incomplete symptom descriptions were excluded. Excel was used to classify the symptoms into three groups: respiratory, digestive and urinary systems. The virus detection information was transformed from the publication [15].

## 3 Results

### 3.1 Expression of ACE2 in normal tissues

**Figure 1:**
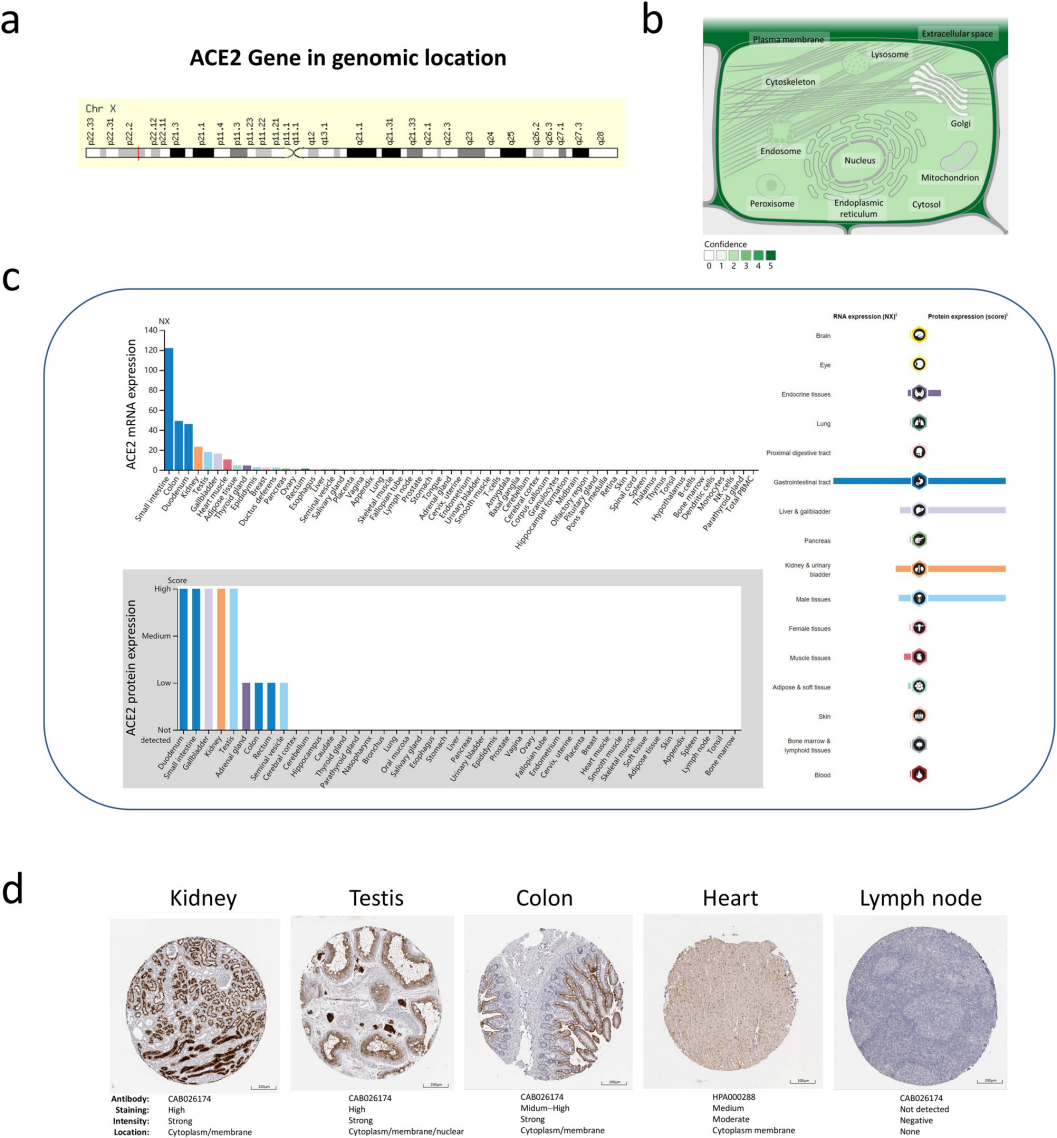
Location and expression of ACE2 in normal tissues. (a) Genomic location of the ACE2 gene obtained from GeneCard. (b) Cellular location of the ACE2 gene (COMPARTMENTS). Confidence scale is color coded, ranging from light green for low confidence to dark green for high confidence. White indicates the absence of localization evidence. (c) The mRNA (left side top, Consensus dataset) and protein (left side down) expressions of ACE2 in normal tissues from the HPA. Color-coding is based on tissue groups, each consisting of tissues with common functional features. (d) Respective images of normal tissue with IHC staining of ACE2 protein in the HPA (scale 200 µm). ACE2: angiotensin-converting enzyme 2; HPA: Human Protein Atlas.

**Figure 2:**
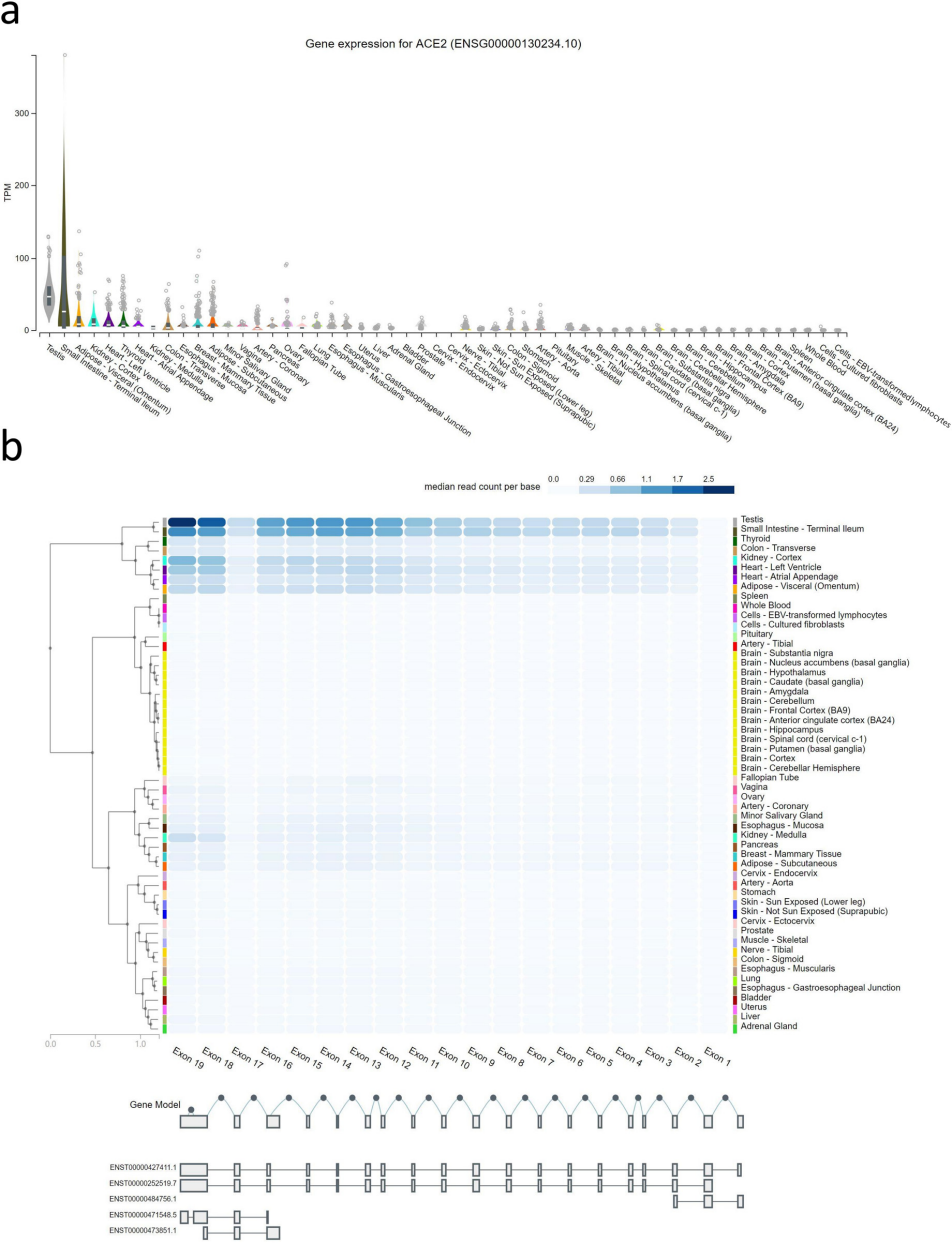
Gene expression of ACE2 in normal tissue (GTEx). (a) Gene expression of ACE2 (ENSG00000130234.10). (b) Exon expression of ACE2 (source: HGNC symbol; Acc: HGNC:13557). ACE2: angiotensin-converting enzyme 2.

GeneCard showed that the ACE2 gene was located in the X chromosome (Xp22.2), encoding a type I transmembrane glycoprotein composed of 805 amino acid residues (Figure 1a). Subcellular locations from COMPARTMENTS revealed confidence 5 in the plasma membrane and extracellular space, confidence 2 in the cytoskeleton, mitochondrion, peroxisome, nucleus, endoplasmic reticulum, endosome, cytosol and lysosome and only confidence 1 in the Golgi apparatus, respectively (Figure 1b). The Consensus dataset using the HPA showed that the mRNA expression of ACE2 in normal tissues from high to low was in five groups. Tissues in the most expressive groups were the small intestine, colon, duodenum, kidney, testis, gallbladder and heart muscle with a score from 122 to 10. The tissues in the second expressive group were the adipose tissue, thyroid gland, epididymis, ductus deferens, breast, pancreas, rectum, ovary, esophagus, liver, seminal vesicle, salivary gland and placenta with a score from 5 to 1. The tissues in three medium expressive groups were the vagina, lung, appendix, skeletal muscle, fallopian tube, lymph node, tongue, stomach and prostate with a score from 0.9 to 0.5. The parathyroid gland, bone marrow, monocytes, B-cells, NK-cells, dendritic cells and total peripheral blood mononuclear cell had no expression of ACE2, and others in the low expressive group had a score from 0.4 to 0.1 (Figure 1c). Immunohistochemical analysis confirmed that ACE2 was highly expressed in the duodenum, gallbladder, kidneys, small intestine and testis, and there was a low expression in the adrenal glands, colon, rectum and seminal vesicles (Figure 1c and 1d). The ACE2 gene has 19 exons, and its expression level showed the same trend as in the HPA which was verified using GTEx (Figure 2).

### 3.2 Expression and prognostic value of ACE2 in malignant tumors

**Figure 3:**
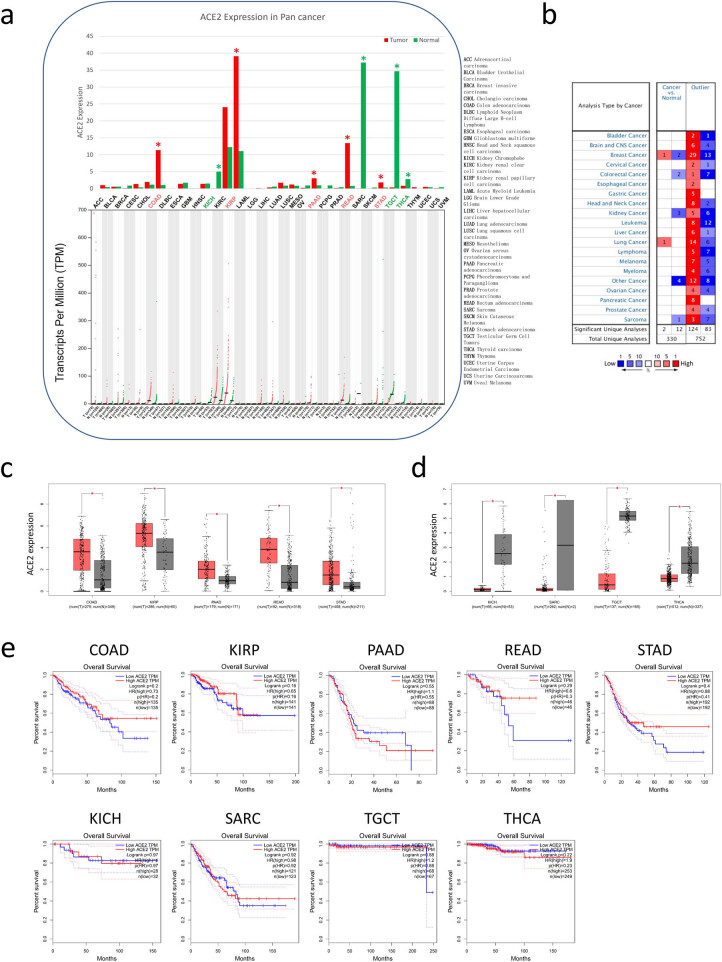
Expression and prognostic value of ACE2 in pan-cancer. Expressions of ACE2 were evaluated in pan-cancer compared with normal tissue from the GEPIA (a) and ONCOMINE (b). ACE2 was overexpressed in five cancer types (c) and decreased in four cancer types (d). The prognostic value of ACE2 in nine cancer types from the GEPIA (e). *P < 0.05. ACE2: angiotensin-converting enzyme 2; GEPIA: Gene Expression Profiling Interactive Analysis.

**Figure 4:**
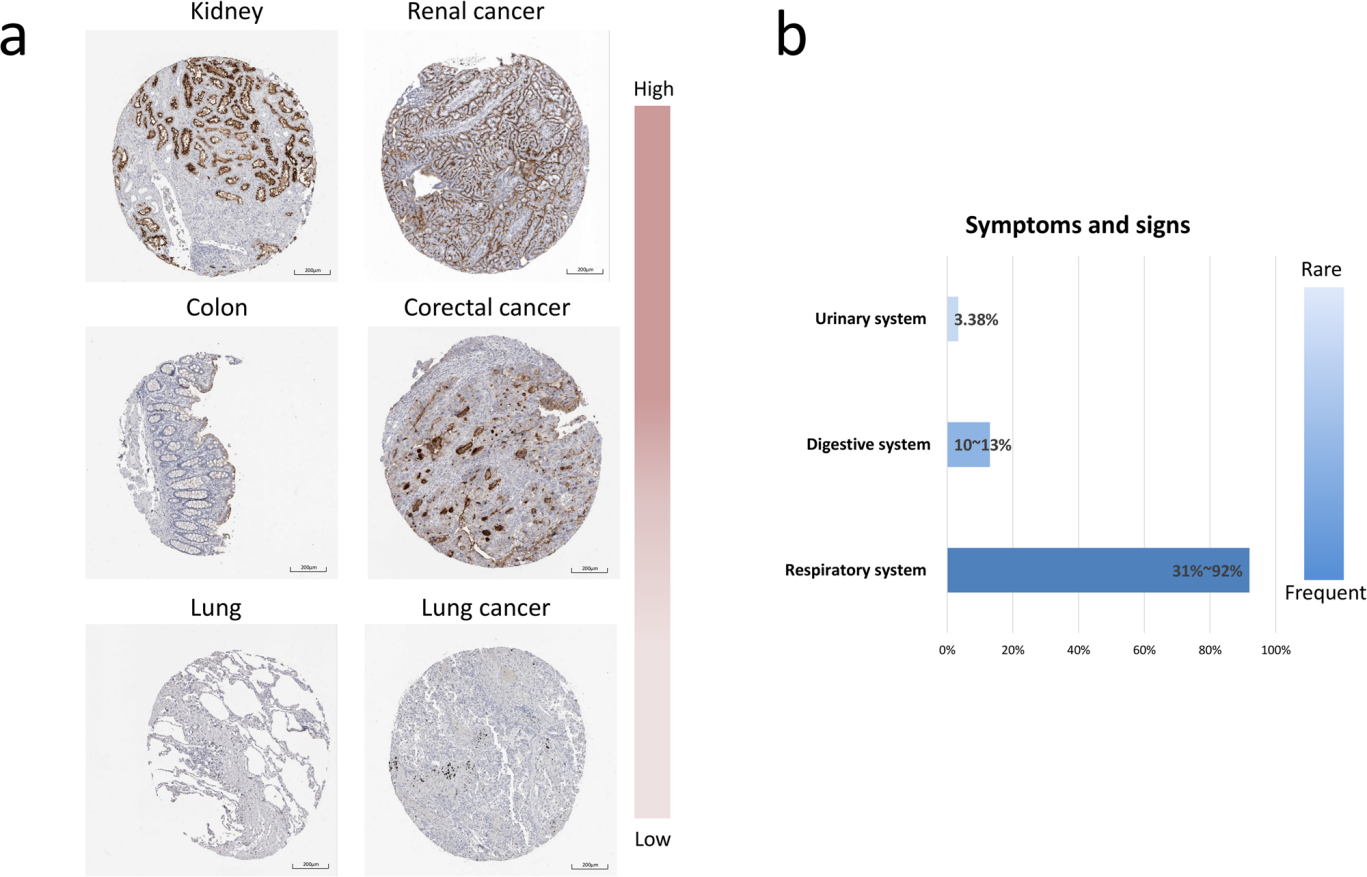
Differences of ACE2 expression and clinical symptoms. (a) Respective images of renal cancer, colorectal cancer and lung cancer compared with normal tissue with IHC staining of ACE2 protein (CAB026174) in the HPA (scale 200 µm). (b) Frequency of three-system related symptoms of COVID-19 patients infected with SARS-CoV-2. ACE2: angiotensin-converting enzyme 2; HPA: Human Protein Atlas; COVID-19: Coronavirus Disease 2019; SARS-CoV-2: severe acute respiratory syndrome coronavirus 2.

Using the GEPIA dataset, we compared the mRNA expression of ACE2 between cancer and normal samples. The results indicated that almost all cancer tissues can express ACE2, and the highest expression level was in renal cancer (Figure 3a). The mRNA expressions of ACE2 were significantly overexpressed in colon adenocarcinoma, kidney renal papillary cell carcinoma, pancreatic adenocarcinoma, rectum adenocarcinoma (READ) and stomach adenocarcinoma (STAD) (Figures 3a and c and 4a). There were significantly lower expressions in kidney chromophobe, sarcoma, testicular germ cell tumors and thyroid carcinoma than those in normal tissues (Figure 3a and d). The gene expressions of ACE2 in cancers and those in normal samples were verified by using ONCOMINE databases (Figures 3b and 4a). ACE2 expressions in lung cancer and breast cancer were upregulated compared with normal tissues. After that, we investigated prognosis value of ACE2 using the GEPIA databases in pan-cancer. Patients in survival analysis were divided into two groups using median ACE2 expression (high vs low expression) and assessed using an overall Kaplan–Meier plot. There were no significant differences in overall survival (OS) between the two ACE2 expression levels. But it seemed that the high ACE2 expression group with READ and STAD had the trend of better OS compared with the low ACE2 expression group (Figure 3e).

### 3.3 Malignant tumors in COVID-19 patients infected with SARS-CoV-2

Studies recently published describing the clinical characteristics of COVID-19 due to SARS-CoV-2 were screened and analyzed. Overall, six studies that evaluated patients’ malignant tumor status were enrolled [12,13,14,16,17,18]. A total of 3,421 cases in China have been confirmed in these studies. Among them, 43 cases (1.26%) had malignant tumor coexisting conditions. Among cancer patients, men and lung cancer patients were more likely to have COVID-19 (Supplementary Table 1). The rate of severe events for malignant tumor patients was 39.02% (16/41), while the rate of severe events for all patients was 10.79% (194/1,798) (Table 1). The clinical symptoms and signs due to COVID-19 were divided into three groups of systems: respiratory, digestive and urinary systems; there were two studies that described symptoms related to all the three systems (Table 2 and Figure 4b). Respiratory symptoms and signs with fever (91.98%), cough (68.78%) and dyspnea (31.22%) were the most frequent in the range from 31% to 92%. Digestive system related symptoms and signs were abdominal pain with/without diarrhea (8.02%) and nausea with/without vomiting (8.02%). We used AKI to represent the urinary system damage, which had the lowest frequency of 3.38%. The ACE2 protein expressions with IHC staining obtained from the HPA were very weak in the lungs, medium in the colon and high in the kidneys, and the same trend was found in the cancer tissues (Figure 4a).

**Table 1:** Number and severity of malignant tumor patients infected with SARS-CoV-2

References	Total case (%)	Malignant tumor (%)	Severe events of tumor patients		Total severe events (%)
			Yes (%)	No (%)	
Dawei Wang et al. (2020)	138	10 (7.25)	4 (40.00)	6 (60.00)	36 (26.09)
Lei Chen et al. (2020)	29	1 (3.45)	NA	NA	14 (48.28)
Nanshan Chen et al. (2020)	99	1 (1.01)	NA	NA	NA
Chaolin Huang et al. (2020)	41	1 (2.44)	0	1	13 (31.71)
Jing Yu et al. (2020)	1,524	12 (0.79)	3 (25.00)	9 (75.00)	NA
Wenhua Liang et al. (2020)	1,590	18 (1.13)	9 (50.00)	9 (50.00)	131 (3.83)
Total	3,421	43 (1.26)	16 (39.02)^a^	25 (60.98)^b^	194 (10.79)^c^

^a^16/41.

^b^7/41.

^c^194/179.

**Table 2:** Representative three-system related symptoms of COVID-19 patients infected with SARS-CoV-2

Symptoms and signs	Dawei Wang et al. (2020)		Nanshan Chen et al. (2020)		Total	
	*n* = 138	(%)	*n* = 99	(%)	*n* = 237	(%)
Respiratory symptoms and signs (31–92%)						
Fever	136	(98.55)	82	(82.83)	218	(91.98)
Cough	82	(59.42)	81	(81.82)	163	(68.78)
Dyspnea	43	(31.16)	31	(31.31)	74	(31.22)
Digestive symptoms and signs (8–13%)						
Abdominal pain, diarrhea	17	(12.32)	2	(2.02)	19	(8.02)
Nausea, vomiting	18	(13.04)	1	(1.01)	19	(8.02)
Urinary symptoms and signs (3.38%)						
AKI	5	(3.62)	3	(3.03)	8	(3.38)

AKI, acute kidney injury; COVID-19, Coronavirus Disease 2019; SARS-CoV-2, severe acute respiratory syndrome coronavirus 2.

## 4 Discussion

SARS-CoV-2 infection is a serious public health problem. As the number of cases has been increasing and as it spreads quickly, people keep a close eye on the future development of this disease. ACE2 as the receptor of SARS-CoV was recognized to be also necessary for the cells infected by SARS-CoV-2, and *in vitro* experiments proved that cells without the ACE2 receptor were not infected by the virus [6,7,9]. Some proposed that the reproduction rate of SARS-CoV-2 is higher than that of SARS-CoV [19]. ACE2 protein is closely related to the entry of this virus, and the distribution of this receptor might reflect the susceptibility to this virus and virus replication. However, the impact of ACE2 on SARS-CoV-2 susceptibility and the situation of malignant tumor patients in this outbreak are unclear. So, it is important to understand the expressions of ACE2 in different tissues and cancers. In our study, results showed that ACE2 was found in almost all the normal tissues, indicating that all the organs were potentially infected. The virus was detected in different samples not only in nasopharyngeal or oropharyngeal swabs but also in stool [15]. There is a possibility of transmission of this virus by the fecal-oral route. Wang et al. had reported ten cases (7.2%) with acute cardiac injury in 138 hospitalized patients with SARS-CoV-2 infection [14]. The expression of ACE2 was also detected in the heart using the mRNA level, so acute cardiac injury might be caused by the virus attacking the heart. Multiple organ functions should be given attention in clinical treatments.

Based on the ACE2 protein expression level, the kidney had a high expression, and the colon in digestive organs had a medium expression. The lungs in the respiratory system had a very low basic expression of mRNA. But SARS-CoV-2 is transmitted mainly through the respiratory tract, and the lungs become the main target of the SARS-CoV-2 attack with the respiratory symptoms being the most frequent in COVID-19. Holshue et al. detected the virus four times in different samples from one COVID-19 patient showing that the initial respiratory specimens (nasopharyngeal and oropharyngeal swabs) obtained were positive four times with a higher Ct value. The digestive system related samples (stool) had only one positive. All the urinary system related samples (urine) were negative [15]. These results suggest that the symptoms might be consistent with virus detection levels, indicating that the more severe the symptoms, the more the virus replicates inducing damage. But the severity of symptoms is not related to the ACE2 expression level. Yu et al. found that vimentin had direct interaction with SARS-CoV spike protein during viral entry in SARS-CoV infection, which might also influence the susceptibility [20]. The SARS-CoV-2 seems more likely to invade tissues with low expression of ACE2. This interesting phenomenon may be caused by the polybasic furin type cleavage site present at the S1–S2 junction in the SARS-CoV-2 spike protein, which is not present in SARS-CoV [21]. Studies on SARS have shown that inserting a multivalent cleavage site in the S1–S2 region of SARS-CoV will result in a moderate but significant increase in fusion activity, which may lead to increased virus entry in low-density ACE2 expressing tissues [22].

Moreover, malignant tumor patients are usually weaker and may be more severely affected by this outbreak. Our results showed that 4.23% of 307 cases had malignant tumor coexisting conditions. The rate of intensive care unit (ICU) admission of COVID-19 patients with malignant tumors (36.36%) showed a higher trend than that for all patients (30.9%). But there was no statistically significant difference. The number of tumor patients was insufficient in the included studies, and further studies are needed for confirmation. The susceptibility of malignant tumor patients and the intensity degree differences could be evaluated by ACE2 expression exploration. The results indicated that almost all cancer tissues can express ACE2, suggesting that all the tumor patients are susceptible to this new SARS-CoV-2. ACE2 expressions in some cancer types such as gastrointestinal tumor and lung cancer were upregulated. Changes in expression levels may affect the susceptibility and disease severity. One of the main causes of death in patients with SARS-CoV-2 is ARDS. The relationship between ACE2 and ARDS has attracted much attention from researchers. Imai et al. found that ACE2 gene knockout in a mice model resulted in increased vascular permeability and increased pulmonary edema, eventually leading to ARDS. However, the use of ACE2 inhibitor significantly reduced the occurrence of ARDS in the mice model [23]. Further research showed that infection of SARS-CoV caused downregulation of ACE2 expression in lung tissues *in vivo* and *in vitro*, which might induce ARDS [24,25]. These results suggested that the expression of ACE2 in mice has a protective effect on the occurrence of ARDS, and changes in ACE2 expression levels are closely related to the severity of this disease. SARS-CoV downregulated ACE2 in the model of respiratory failure, and the viral spike protein induced TNF-alpha-converting enzyme-dependent shedding of the ACE2 ectodomain and led to tissue damage [26]. Increasing ACE2 expression is a potential strategy to prevent ARDS in COVID-19 patients. We should carefully use treatments such as ACE2 inhibitors.

## 5 Conclusion

In summary, our study found ACE2 expressions in different tissues. The normal tissues of the kidneys, duodenum, intestine, gallbladder and testis had the highest expression, followed by the colon, rectum and seminal vesicles. The lungs had very low expression suggesting that low expression of ACE2 is enough for the infection of SARS-CoV-2. ACE2 expressions were upregulated in renal cancer, gastrointestinal tumor and lung cancer. There was no significant difference in severity of COVID-19 in malignant tumor patients. In particular, organ damage was likely consistent with virus detection levels but negatively related to the protein expression level. The mechanism of virus entry into human cells may require other receptors or cofactors. These findings may help to develop anti-SARS-CoV-2 agents.

## Abbreviations

COAD: Colon adenocarcinoma
COVID-19: Coronavirus disease 2019
ACE2: Angiotensin-converting enzyme 2
AKI: Acute kidney injury
ARDS: Acute respiratory distress syndrome
GEPIA: Gene expression profiling interactive analysis
GTEx: Genotype tissue expression
HPA: Human protein atlas
ICTV: International committee on taxonomy of viruses
ICU: Intensive care unit
IHC: Immunohistochemistry
KICH: Kidney chromophobe
KIRP: Kidney renal papillary cell carcinoma
NCIP: Novel coronavirus
NX: Normalized eXpression
OS: Overall survival
PAAD: Pancreatic adenocarcinoma
READ: Rectum adenocarcinoma
SARC: Sarcoma
SRAS-CoV2: Severe acute respiratory syndrome coronavirus 2
STAD: Stomach adenocarcinoma
TACE: Tumor necrosis factor-alpha-converting enzyme
TCGA: The cancer genome atlas
TGCT: Testicular germ cell tumors
THCA: Thyroid carcinoma
